# Autism beyond diagnostic categories: characterization of autistic phenotypes in schizophrenia

**DOI:** 10.1186/s12888-015-0494-x

**Published:** 2015-05-13

**Authors:** Anne Kästner, Martin Begemann, Tanja Maria Michel, Sarah Everts, Beata Stepniak, Christiane Bach, Luise Poustka, Joachim Becker, Tobias Banaschewski, Matthias Dose, Hannelore Ehrenreich

**Affiliations:** 1 Clinical Neuroscience, Max Planck Institute of Experimental Medicine, Hermann-Rein-Str.3, 37075 Göttingen, Germany; 2 DFG Research Center for Nanoscale Microscopy and Molecular Physiology of the Brain (CNMPB), Göttingen, Germany; 3 Department of Psychiatry, Institute for Clinical Research, University of Southern Denmark, Odense, Denmark; 4 Child and Adolescent Psychiatry and Psychotherapy, Central Institute of Mental Health, Mannheim, Germany; 5 kbo-Isar-Amper-Klinikum Taufkirchen, Taufkirchen (Vils), Germany

**Keywords:** Autism spectrum disorders, Positive and negative syndrome scale, Autism diagnostic observation schedule, Diagnostics, Autism quotient, Empathy quotient, Adults

## Abstract

**Background:**

Behavioral phenotypical continua from health to disease suggest common underlying mechanisms with quantitative rather than qualitative differences. Until recently, autism spectrum disorders and schizophrenia were considered distinct nosologic entities. However, emerging evidence contributes to the blurring of symptomatic and genetic boundaries between these conditions. The present study aimed at quantifying behavioral phenotypes shared by autism spectrum disorders and schizophrenia to prepare the ground for biological pathway analyses.

**Methods:**

Specific items of the Positive and Negative Syndrome Scale were employed and summed up to form a dimensional autism severity score (*PAUSS*). The score was created in a schizophrenia sample (N = 1156) and validated in adult high-functioning autism spectrum disorder (ASD) patients (N = 165). To this end, the Autism Diagnostic Observation Schedule (ADOS), the Autism (AQ) and Empathy Quotient (EQ) self-rating questionnaires were applied back to back with the newly developed *PAUSS.*

**Results:**

*PAUSS* differentiated between ASD, schizophrenia and a disease-control sample and substantially correlated with the Autism Diagnostic Observation Schedule. Patients with ADOS scores ≥12 obtained highest, those with scores <7 lowest *PAUSS* values. AQ and EQ were not found to vary dependent on ADOS diagnosis. ROC curves for ADOS and *PAUSS* resulted in AuC values of 0.9 and 0.8, whereas AQ and EQ performed at chance level in the prediction of ASD.

**Conclusions:**

This work underscores the convergence of schizophrenia negative symptoms and autistic phenotypes. *PAUSS* evolved as a measure capturing the continuous nature of autistic behaviors. The definition of extreme-groups based on the dimensional *PAUSS* may permit future investigations of genetic constellations modulating autistic phenotypes.

## Background

Autistic phenotypes transcend diagnostic categories. Sub-threshold deficits in social communication and restricted interests which do not meet formal criteria for an autism spectrum disorder (ASD) can be found in the general population [1-4]. This supports the dimensional nature of autistic traits. If surpassing a certain severity threshold, autistic behaviors may become clinically relevant. Different expressions of autistic phenotypes can also be observed in patients with psychiatric diagnoses other than ASD [5].

Particularly, the very heterogeneous diagnostic category of schizophrenia harbors a distinct subgroup of individuals with severe autistic features. Historically, autism spectrum disorders (ASD) and schizophrenia were considered to be intimately related [6-9]. Based on early epidemiological studies, they were later conceptualized as distinct diagnostic entities due to e.g. presenting characteristics, course and age of onset [10]. However, boundaries between psychiatric diagnoses begin to blur. DSM-IV lists 522 criteria for diagnosing 201 distinct psychiatric conditions [11,12]. Hence, several symptoms constitute criteria for more than one disorder. Also, genetic risk factors simultaneously associate with several psychiatric diseases [13-15]. This reflects their modulating individual behavioral phenotypes instead of diagnostic categories.

In fact, many patients fulfill criteria for both schizophrenia and an autism spectrum disorder [16-18]. In both conditions, neuropathological findings support developmentally altered synaptic connectivity [19-21]. Variations in synaptic genes contribute to the susceptibility to both disorders [14,22-24]. Neurodevelopmental abnormalities typical for children with ASD such as a delay in motor development, impaired receptive language as well as relationship and adjustment difficulties are also found to prevail in individuals later diagnosed with schizophrenia [25]. Along the same lines, recent studies convincingly demonstrate that childhood-onset schizophrenia is preceded by an ASD diagnosis in 30%-50% of the cases [25]. Most importantly, however, among those suffering from schizophrenia, some exhibit a prominent autistic phenotype while psychotic symptoms are less prominent [26,27]. This autistic subgroup of schizophrenic patients can be characterized by difficulties in social interaction, communication, emotion processing, and motor abnormalities [27,28]. Schizophrenia patients predominantly suffering from negative symptoms obtain high scores on the Autism Diagnostic Observation Schedule (ADOS) [26]. Taken together, the strong phenotypic relationship suggests overlapping disease mechanisms involved in ASD and at least a subset of schizophrenia patients.

The present study has been designed to provide a dimensional measure for investigations of biological pathways common to ASD and the autistic subgroup of schizophrenia patients. Autistic phenotypes were characterized in schizophrenia patients using items from the Positive and Negative Syndrome Scale (PANSS) which has been assessed in the frame of the GRAS data collection [29,30]. The high internal consistency of all individual autism variables derived from PANSS encouraged their aggregation to form a continuous autism severity score (PANSS autism severity score, *PAUSS*). As the selected items had not been intended to assess autism-relevant behaviors, they were subsequently validated in a sample of high-functioning ASD patients, including patients with Asperger’s disorder and autistic disorder with average intellectual functioning (validation sample). By demonstrating a high convergence of the Autism Diagnostic Observation Schedule (ADOS) and the *PAUSS*, we illustrate that the latter is suitable for the assessment of the severity of autistic behaviors in schizophrenia and high-functioning autism.

## Methods

### Schizophrenia (GRAS) sample

#### Participants

The present study was approved by the ethics committee of the Georg-August-University (master committee) and local internal review boards of collaborating centers. All patients gave written informed consent. Schizophrenia diagnoses were made based on DSM-IV-TR criteria [11]. Detailed phenotyping of the GRAS sample [29-31] contained the Positive and Negative Syndrome Scale (PANSS) [32]. Data analyses were based on 1156 schizophrenia patients (N = 770 male, N = 386 female; referred to as ‘GRAS sample’, for detailed information on the GRAS data collection see [30]).

#### Operationalization of autistic phenotypes in the GRAS sample –The PANSS autism severity score (PAUSS)

**Figure 1:**
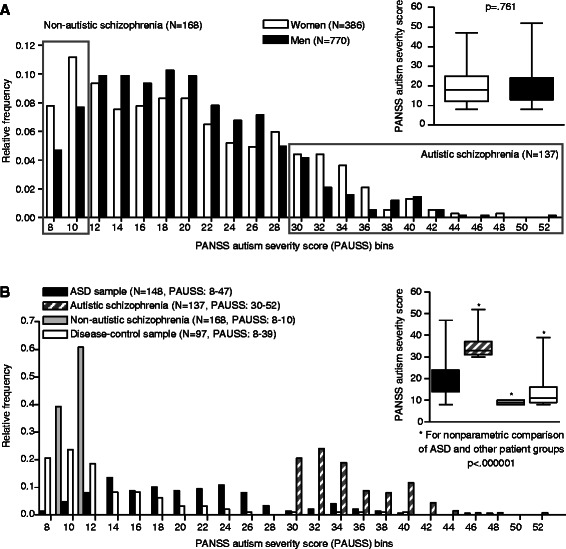
Distribution of the PANSS autism severity score (*PAUSS*) in the schizophrenia, ASD and disease-control samples. **(A)** Relative frequency distributions of the *PAUSS* bins in the schizophrenic GRAS sample by gender are shown. The first and last percentile of the distribution (‘autistic schizophrenics’ and ‘non-autistic schizophrenics’) is contrasted with respect to sociodemographic and clinical characteristics in Table 4. **(B)** Comparison of the relative frequency distribution of the *PAUSS* in the GRAS sample and the validation sample (split into ASD sample and disease-control sample). Inset figures show means ± SD. All p-values were obtained from Mann-Whitney U-tests.

From the thorough phenotype information available in the GRAS sample [30], items indicative of autistic behavior (covering all three symptom domains according to DSM-IV-TR) [11] were selected from PANSS [32]. The PANSS is a standardized third-party clinical observation tool, well-evaluated and widely applied to assess positive, negative, general psychopathology symptom severity in schizophrenia [32]. All raters (psychologists, psychiatrists) participating in data acquisition for the GRAS collection over the last 10 years had intensive trainings, regular consensus meetings, and repeated interrater reliability testings over the whole GRAS examination book [30], including PANSS. The severity scoring of PANSS items ranges from 1 (definition does not apply) to 7 (severe dysfunction). To cover the ASD diagnostic domain of *difficulties in social interaction*, items 1 (‘blunted affect’); 3 (‘poor rapport’) and 4 (‘social withdrawal’) of the PANSS negative subscale were used. *Difficulties in communication* were measured employing items 5 (‘difficulties in abstract thinking’) and 6 (‘lack of spontaneity and flow of conversation’) of the PANSS negative subscale. The third diagnostic symptom cluster assessing *limited, repetitive and stereotypic patterns of behavior* was accounted for by using item 5 (‘mannerism’) and 15 (‘preoccupation’) of the PANSS general subscale and item 7 of the negative subscale (‘stereotyped thinking’). All individual items were summed up to form the PANSS autism severity score (*PAUSS*, range 8 to 56). Higher *PAUS* scores represent a higher severity of the autism phenotype. Extreme-groups (autistic and non-autistic schizophrenia individuals) include the first (*PAUSS* 8-10) and the last percentile (*PAUSS* 30-52) of the *PAUSS* distribution (Figure 1A and B).

### ASD sample

#### Participants

The study was approved by the ethics committee of the Georg-August-University, Göttingen. All participants gave written informed consent. A total of 260 adult patients with an established DSM-IV-TR ASD diagnosis and 5 cases suspected to suffer from ASD by a health care professional (practicing clinical psychologists, psychiatrists or specialized German outpatient clinics personnel) were included (N = 178 male, N = 87 female). They were recruited from September 2011 to October 2014 via public announcements and collaborations with specialized autism centers all over Germany. Further prerequisites for inclusion in the present study were an IQ ≥ 75 and expressive language skills allowing for the conductance of a semi-structured interview.

#### Measures and procedure of the PAUSS validation

Due to the lack of an established diagnostic routine for ASD in adults in Germany, reliability of ASD diagnoses was expected to vary depending on the practitioners’ knowledge and experience. Therefore, DSM-IV-TR ASD diagnoses were confirmed or excluded for all subjects applying the following procedure: A psychologist (AK) and a psychiatrist (MB) with together more than 20 years of clinical experience, both with special training in diagnostics of ASD and relevant differential diagnoses, examined essentially all patients. Participants were welcomed by both examiners. A friendly and casual atmosphere was created, in which spontaneous social-communicative behavior could be observed. Basic information about the medical, social and family history was recorded. This initial phase was followed by the conductance of module 4 of the Autism Diagnostic Observation Schedule (ADOS) [33,34]. Module 4 has been developed for adolescents and adults with fluent speech and has good criterion-related validity [26,33,35]. The administration of ADOS module 4 included all standard and optional activities. Both ADOS raters of the present study had undergone a special training to guarantee standardized administration and scoring. Additionally, a semi-structured interview for the assessment of autism-relevant developmental history (motor, cognitive and speech development), current daily functioning and quality of relationships was performed and used for supporting the autism diagnosis (for a description of the interview questions see Table 1; detailed psychometrical analysis and validation of this new instrument will be provided elsewhere)**.** The *PAUSS* items were rated according to the PANSS manual. Moreover, a 33-item version of the Autism Quotient (AQ) and the Empathy Quotient (EQ) self-rating questionnaires were administered [1,36,37]. Four subtests of the German version of the Wechsler Adult Intelligence Scale revised (WAIS-R) were completed to estimate verbal and performance IQ (comprehension, similarities, picture completion and block design) [38]. The Global Assessment of Functioning (GAF) was scored to determine the global functional status of the participants [11]. ASD diagnoses were confirmed or excluded according to DSM-IV-TR criteria and to the guideline of the United Kingdom’s National Institute for Health and Care Excellence (NICE). The diagnosis was always based on the consensus of the two investigators.

**Table 1:** **The semi-structured interview for the assessment of developmental history, current daily functioning and quality of relationships**

Given are the interview questions; a detailed psychometrical evaluation of the instrument will be published elsewhere	
**Part 1: EARLY DEVELOPMENT**	
***Self regulation***	Has anyone ever told you that you cried a lot when you were an infant and that it was almost impossible to calm you down?
	Has anyone ever told you about feeding problems when you were an infant?
	When you were a toddler, did you avoid eating certain things?
	Has anyone ever told you that you had difficulties falling asleep or that you could not sleep through the night?
***Motor development***	How old were you when you started walking?
	When you were a child (between 4 and 5 years old), did you enjoy playing ball, doing rope skipping or other things involving physical exercises?
	Which of the following sports did you excel in: Endurance runs, track and field athletics, climbing, dancing, artistic gymnastics, soccer, basketball, volleyball, team handball, swimming?
	Have you ever practiced any kind of team sports for at least a year? When was that?
	Did you like to do things involving fine motor function, e.g. playing Mikado, building card houses, when you were a child?
	Have you ever received occupational therapy, hippotherapy or comparable measures? For how long?
***Speech development***	At how many months did you speak first words?
	At how many months did you speak first two-word-sentences?
	Has anybody ever told you that there was something special about the way you talked?
	Have you ever had difficulties understanding what other people wanted to tell you?
	Have you often been told that you never listened/that you often seemed absent-minded?
	Has anyone ever told you that you had a very elaborate vocabulary when you were a child?
	Have you ever been treated by a speech therapist?
**Part 2: DEVELOPMENT & EDUCATION**	
***Academic performance (proxy for cognitive development)***	How old were you at school enrollment?
	Have you ever had to stay down a grade? If yes, which grade?
	Have you ever skipped a grade?
	Have you ever had difficulties concentrating?
	Was there a particular subject or topic that you performed particularly well or poorly at?
***Quality of social contacts and activities in childhood, adolescence and early adulthood***	How did you mostly occupy yourself during the school breaks?
	How were the kindergarten times for you (age 3 to 6 years)?
	Was in the kindergarten something particularly difficult/unpleasant?
	What did you like to play in kindergarten times (age 3 to 6 years)?
	With whom did you play in kindergarten times (age 3 to 6 years)?
	How were the days in elementary school for you (age 6 to 10 years)?
	Was there something particularly difficult/unpleasant?
	Did you have friends when you were 6 to 10 years old? How many?
	What did you like to do in your leisure time when you were 6 to 10 years old?
	How were the high school days for you (age 10 to 18 years)?
	Did you have friends when you were 10 to 18 years old? How many?
	What did you like to do in your leisure time when you were 10 to 18 years old?
	Did you suffer from not having friends or from having few friends?
**Part 3: CURRENT STATUS**	
***Daily routines***	Now I am interested in your daily routine. Please describe it to me.
	Are there any rigid routines, for instance exact and narrow timing of the steps involved in the dental hygiene or getting dressed?
	Do certain things belong to a particular place?
	Do you prepare lists or schedules? Do you enjoy sorting things? Do these lists/schedules serve a certain purpose for you or others?
	On a scale from 0 to 10, how unpleasant are new situations for you?
	Why are new situations unpleasant?
	What do you like to do in your leisure time?
	On average, how many hours do you spend with a particular topic?
	Do you like to engage in certain mental routines like counting steps of a staircase or extracting roots?
	Are there any numbers you particularly like, dislike or that you pay attention to?
***Relationships to others***	Who are the most important people in your life?
	Is it difficult for you to get into contact with others?
	Why is it difficult to get into contact with others?
	Is it difficult to maintain a relationship over a longer period of time?
	Why is it difficult to maintain a relationship over a longer period of time?
	Do you experience misunderstandings with others very often?
	What do you think are the reasons for these misunderstandings?
	Would you like to have more contact to others?

As PANSS had not been developed and evaluated for the assessment of autism-relevant behaviors, construct and criterion-related validity of the *PAUSS* were evaluated. To provide convergent validity, ADOS module 4 was scored based on the ‘original algorithm’ relying on the social interaction and communication domain [33]. Additionally, criterion-related validity referring to the quality of differentiation between autistic and non-autistic individuals should be assessed for the *PAUSS*. It is important to note, that always one of the two examiners administered and scored the ADOS while the other conducted the semi-structured interview and rated the *PAUSS* items. Thus, both ratings were independent of each other and the clinical diagnosis.

### Disease-control sample

Upon exclusion of an ASD diagnosis, alternative DSM-IV-TR diagnoses were made based on the clinical information obtained during the interviews and the medical reports available. For economic reasons, only a rough classification into major DSM-IV-TR disease categories was possible for these cases. All of them were included in a so-called disease-control sample. In the present study, the ASD sample is presented as one diagnostic entity (no distinctions between Asperger’s and autistic disorders made) along a continuous severity dimension.

### Statistical analyses

**Figure 2:**
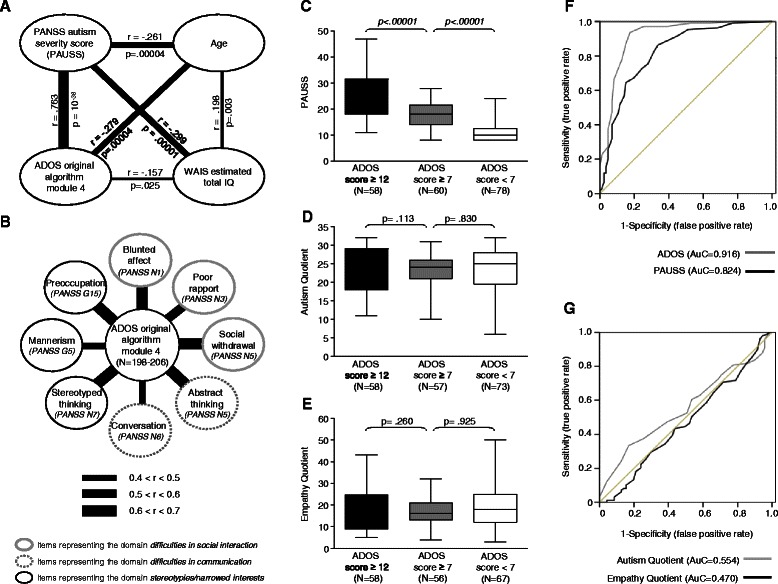
Validation of the *PAUSS*. **(A)** Intercorrelations of the *PAUSS* and ADOS with age and WAIS estimated total IQ (Spearman rank) **(B)** Intercorrelations of individual *PAUSS* items (Spearman rank) and the Autism Diagnostic Observation Schedule (ADOS). **(C)** Significant differences were obtained for the *PAUSS* by ADOS-diagnosis group (ADOS ≥ 12: autism; ADOS ≥ 7: autism spectrum; ADOS < 7: no autism). **(D, E)** No significant differences were found for AQ and EQ by ADOS-diagnosis group. **(F)** Receiver Operating Characteristic curves for *PAUSS* and ADOS illustrate high Area under the Curve (AuC) statistics and thus high predictive power of both instruments. **(G)** Receiver Operating Characteristic curves for AQ and EQ illustrate low Area under the Curve (AuC) statistics and thus low predictive power of both instruments. P-values were obtained from analysis of covariance with age and IQ as covariates.

All statistical analyses were conducted with SPSS (SPSS Statistics for Windows, Version 17.0. Chicago: SPSS Inc.) or GraphPad prism 4.00 for Windows (GraphPad Software, San Diego California USA, www.graphpad.com). Spearman rank correlation coefficients are reported for autism variables in the GRAS sample (Table 2) and *PAUSS*, ADOS, age and IQ (Figure 2A) as well as individual *PAUSS* items and ADOS (Figure 2B). In the present study, criterion-related validity refers to the degree to which ADOS and *PAUSS* ratings are in agreement with the clinical diagnosis of having ASD or not. For the ADOS, the diagnoses based on the original ADOS algorithm module 4 were used: Autism is diagnosed if a subject scores ≥ 12; autism spectrum disorder is diagnosed when a score of ≥ 7 is obtained. Scoring below 7 does not support an ASD diagnosis. ADOS diagnosis group differences in *PAUSS*, AQ and EQ were assessed by analysis of covariance including covariates age and IQ as they correlated substantially with the *PAUSS* (Figure 2C-E). Receiver Operating Characteristic (ROC) curves were calculated to provide information on the sensitivity and specificity of all possible threshold settings for ADOS and *PAUSS* (Figure 2F, G). Sensitivity and specificity at all possible PAUSS cut-offs are provided (Table 3). Area under the Curve (AuC) statistics representing the overall level of agreement between criterion (i.e. clinical ASD diagnosis) and instrument (i.e. ADOS and *PAUSS*) were determined. The higher the AuC (1 = perfect agreement), the higher the probability for a randomly chosen ASD patient to score higher on the respective instrument than a randomly chosen proband without ASD. Group differences (Tables 4, 5, 6) were assessed by Mann-Whitney U tests (continuous variables) or Chi-square/Fisher’s exact test (categorical variables). All p-values are two-sided. The respective statistical procedures applied are additionally mentioned in Table footnotes and Figure legends. Bonferroni multiple testing corrections were performed as indicated there.

**Table 2:** **Item-item intercorrelation matrix for individual PAUSS items in the schizophrenic GRAS sample (N = 1159; Cronbach’s alpha: .857)**

	Blunted affect	Poor rapport	Social withdrawal	Abstract thinking	Conversation	Stereotyped thinking	Mannerism
	(PANSS N1)	(PANSS N3)	(PANSS N4)	(PANSS N5)	(PANSS N6)	(PANSS N7)	(PANSS G5)
Poor rapport (PANSS N3)	**.668**						
Social withdrawal (PANSS N4)	**.574**	**.577**					
Abstract thinking (PANSS N5)	**.472**	**.452**	.303				
Conversation (PANSS N6)	**.577**	**.598**	**.573**	**.432**			
Stereotyped thinking (PANSS N7)	**.414**	**.442**	.332	**.400**	.254		
Mannerism (PANSS G5)	.263	.244	.218	.245	.137	.315	
Preoccupation (PANSS G15)	**.490**	**.558**	**.499**	.391	.363	**.523**	.293

*Spearman rank correlation coefficients are shown (N = 1159). All item-item correlations are statistically significant (p-values < .00001). Correlation coefficients ≥ 0.4 are set in boldface.*

**Table 3:** **PANSS autism severity scores and their corresponding sensitivity and specificity values**

PANSS autism severity score	Sensitivity	Specificity	PANSS autism severity score	Sensitivity	Specificity
7.0	1.000	0.000	24.5	0.243	0.938
8.5	0.986	0.206	25.5	0.196	0.938
9.5	0.953	0.289	26.5	0.162	0.948
10.5	0.939	0.443	27.5	0.142	0.948
11.5	0.912	0.526	28.5	0.128	0.948
12.5	0.858	0.629	30.0	0.115	0.959
**13.5**	**0.804**	0.680*	31.5	0.101	0.959
**14.5**	**0.723**	0.711**	32.5	0.095	0.959
15.5	0.696	0.742	33.5	0.074	0.969
16.5	0.635	0.794	34.5	0.054	0.969
17.5	0.608	0.845	36.0	0.034	0.979
18.5	0.534	0.856	37.5	0.027	0.979
19.5	0.500	0.876	38.5	0.020	0.990
20.5	0.446	0.887	42.0	0.014	1.000
21.5	0.385	0.918	46.0	0.007	1.000
22.5	0.351	0.918	48.0	0.000	1.000
23.5	0.297	0.918			

*The cut-off of 15 (14.5) yields a sensitivity of 72.3% and a specificity of 71.1%. **When using a cut-off of 14 (13.5) sensitivity is increased to 80.4% at the cost of a reduction of specificity to 68%.

**Table 4:** **Sociodemographic and clinical comparison of autistic and non-autistic schizophrenics (GRAS sample)**

	Autistic schizophrenia patients ^ a ^		Non-autistic schizophrenia patients ^ a ^	
	Men	Women	Men	Women
	N = 75-80	N = 53-57	N = 86-96	N = 62-72
**Sociodemographic variables**				
Age at examination*, mean* ± *SD, y*^*b*^	42.1 ± 13.2	49.2 ± 14.5	36.3 ± 11.9	41 ± 10.9*
Ethnicity*, No. (%), Caucasian*^*c*^	76 (95)	53 (98.1)	92 (96.8)	71 (98.6)
Years of education*, mean* ± *SD*^*b*^	11 ± 2.7	11.2 ± 2.1	13.4 ± 3.1***	14.2 ± 3.3***
Current occupation*, No. (%), full-time work*^*d*^	5 (6.3)	1 (1.8)	16 (16.8)	11 (15.1)
Marital status, No. (%), *single*^*c*^	71 (88.8)	31 (54.4)	75 (78.9)	32 (43.8)
Children, No. (%), *without*^*c*^	70 (87.5)	28 (49.1)	78 (82.1)	39 (53.4)
Relationship status, No. (%), *no relationship*^*c*^	67 (83.8)	31 (54.4)	68 (71.6)	26 (35.6)
**Clinical variables**				
Age at first episode*, mean* ± *SD, y*^*b*^	26.0 ± 9.2	30.8 ± 12.6	25.8 ± 7.2	29.1 ± 9.8
Duration of disease*, mean* ± *SD, y*^*b*^	16.3 ± 12.9	18.2 ± 14.4	10.6 ± 9.8	12.1 ± 9.6
Chlorpromazine equivalents, *mean* ± *SD*^*b*^	946 ± 863	972 ± 1321	562.4 ± 567*	401.6 ± 489**
Number of hospitalizations, *mean* ± *SD*^*b*^	10.8 ± 13	10.7 ± 11.5	5.7 ± 4.8	5 ± 4.6*
Premorbid intelligence, *mean* ± *SD, IQ*^*e*^	95 ± 15	97 ± 13	108 ± 16***	109 ± 14***
PANSS pos*, mean* ± *SD*^*b*^	19.4 ± 7	19.3 ± 8	9.1 ± 2.9***	8.7 ± 2.5***
PANSS neg*, mean* ± *SD*^*b*^	32.6 ± 4.5	32.4 ± 4.2	8.1 ± 1.2***	8 ± 1.1***
PANSS gen*, mean* ± *SD*^*b*^	50.5 ± 11.2	51.5 ± 10.8	20.7 ± 4.9***	20.7 ± 4.2***
PANSS total*, mean* ± *SD*^*b*^	102.5 ± 19.1	103.5 ± 18.2	37.9 ± 7.4***	37.4 ± 6.1***
GAF*, mean* ± *SD*^*b*^	30 ± 10.9	28.1 ± 9.9	62.2 ± 15.2***	69.2 ± 14.2***

Multiple testing adjusted significances (Bonferroni: p ≤ 0.003) for comparison of ‘autistic schizophrenia patients’ versus ‘non-autistic schizophrenia patients’ by gender are shown: * ≤ 0.001, ** ≤ 0.0001, *** ≤ 0.00001; due to missing data upon phenotyping sample size varies between N = 128-137 in the autistic schizophrenia group and N = 148-168 in the non-autistic schizophrenia group;^*a*^*Compare with Figure*1*: ‘Autistic schizophrenia patients’ score above or equal to the 10*^*th*^*percentile of the PAUSS distribution (PAUSS 30-52), ‘Non-autistic schizophrenia patients’ score below or equal to the first percentile of the PAUSS distribution (PAUSS 8-10);*^*b*^*Mann-Whitney U-Test;*^*c*^*χ*^*2*^*-Test;*^*d*^*Fisher’s exact test;*^*e*^*Premorbid intelligence was estimated by using the ‘Mehrfachwahlwortschatztest’ (MWT,* multiple choice verbal comprehension test).

**Table 5:** **Sociodemographic and clinical comparison of ASD and disease-control samples**

	ASD sample	Disease-control sample				
		Personality disorders	Psychotic disorders	Other psychiatric disorders ^ a ^	No psychiatric disorder	Total disease-control sample
	N = 106-165	N = 19-26	N = 6-13	N = 38-42	N = 17-19	N = 80-100
**Sociodemographic variables**						
Gender, *No. (%), men*^b^	108 (65.5)	21 (80.8)	7 (53.8)	30 (71.4)	12 (63.2)	71 (71)
Age at examination, *mean ± SD, years*^b^	32.2 ± 11	39.3 ± 12.8	32.6 ± 10.1	38.7 ± 14.3	38.1 ± 12	37.7 ± 13.1*
Years of education, *mean ± SD*^b^	15.3 ± 4.4	15.9 ± 4.8	12.1 ± 4	16.4 ± 4.1	17 ± 4.6	16 ± 4.5
Current occupation, *No. (%), full-time work*^b^	39 (23.6)	6 (23.1)	0 (0)	13 (31)	9 (47.4)	28 (28)
Marital status, *No. (%), single*^c^	101 (61.2)	12 (46.2)	8 (61.5)	25 (59.5)	7 (36.8)	52 (52)
Children, *No (%), none*^c^	103 (83.7)	12 (57.1)	6 (85.7)	27 (67.5)	9 (50)*	54 (62.1)**
Relationship status, *No. (%), no relationship*^c^	68 (41.2)	11 (42.3)	5 (38.5)	23 (54.8)	6 (31.6)	45 (45)
**Clinical variables**						
Wechsler Adult Intelligence Scale-Revised, *IQ*						
*subtest comprehension*	116 ± 19	113 ± 13	104 ± 19	114 ± 17	117 ± 14	113 ± 16
*subtest similarities*	115 ± 16	114 ± 13	101 ± 16	120 ± 20	117 ± 15	116 ± 18
*subtest picture completion*	107 ± 21	111 ± 18	98 ± 26	107 ± 21	111 ± 13	108 ± 19
*subtest block design*	109 ± 20	107 ± 18	95 ± 23	110 ± 17	111 ± 17	108 ± 18
*estimated verbal IQ*	115 ± 16	112 ± 10	103 ± 14	116 ± 18	117 ± 12	114 ± 15
*estimated performance IQ*	107 ± 18	106 ± 18	96 ± 21	108 ± 18	111 ± 12	107 ± 17
*estimated total IQ*	111 ± 15	110 ± 12	99 ± 17	112 ± 16	114 ± 11	111 ± 14
GAF, *mean ± SD*^b^	71 ± 16.2	75.7 ± 11.2	52.5 ± 13	74.4 ± 14.8	87.6 ± 5.3***	75.5 ± 15
PANSS autism severity score, *mean ± SD*^b^	20.5 ± 7.7	13.3 ± 3.6***	24.1 ± 9.6	11.4 ± 3.7***	9.8 ± 2.2***	13.3 ± 6.6***
ADOS original algorithm Module 4, *mean ± SD*^b^	11.9 ± 4.2	4.3 ± 4.4***	10 ± 4.8	2.5 ± 2.8***	2.9 ± 2.2***	3.8 ± 3.9***
Autism Quotient (AQ), *mean ± SD*^b,d^	23.5 ± 5.7	24 ± 3.6	20.7 ± 4	23.3 ± 5.7	23.2 ± 5.1	23.4 ± 5.1
Empathy Quotient (EQ), *mean ± SD*^b,e^	17.5 ± 8	17.8 ± 8.3	25.1 ± 8.7	21.2 ± 11.6	15.6 ± 9.3	19.4 ± 17.5

Multiple testing adjusted significances (Bonferroni: p ≤ 0.002) for comparison of each diagnostic group with ASD group are shown: * ≤ 0.001, ** ≤ 0.0001, *** ≤ 0.00001; due to missing data upon phenotyping, sample size varies between N = 186-265 in the total sample; ^a^ *this category includes affective disorders, attention deficit disorder, pervasive developmental disorder not otherwise specified, anxiety disorders, alcohol use disorders;* ^b^ *Mann-Whitney U test;* ^c^ *Chi-square test;* ^d^ *Higher values correspond to more autistic traits;* ^e^ *Higher values correspond to more empathy (i.e. less autistic traits).*

**Table 6:** **Sociodemographic and clinical comparison of ASD and disease-control samples by gender**

	ASD sample			Disease-control sample ^ a ^		
	Men	Women	p-value	Men	Women	p-value
	N = 70-107	N = 37-58		N = 58-71	N = 21-29	
**Sociodemographic variables**						
Age at examination, *mean ± SD, years*^b^	31.3 ± 11.3	34.1 ± 10.3	.050	37.6 ± 13.9	38 ± 11.2	.747
Years of education, *mean ± SD*^b^	14.6 ± 4.1	16.5 ± 4.8	.040	15.9 ± 4.4	16.2 ± 4.8	.529
Current occupation, *No. (%), full-time work*^b^	25 (23.4)	14 (24.1)	1.00	18 (25.4)	10 (34.5)	.462
Marital status, *No. (%), single*^c^	67 (62.6)	34 (58.6)	.620	40 (56.3)	12 (41.4)	.193
Children, *No (%), none*^c^	68 (86.1)	35 (81.4)	.602	42 (66.7)	12 (50)	.216
Relationship status, *No. (%), no relationship*^c^	47 (43.9)	21 (36.2)	.408	35 (49.3)	10 (34.5)	.192
**Clinical variables**						
Wechsler Adult Intelligence Scale-Revised, *IQ*						
*subtest comprehension*	117 ± 20	115 ± 18	.396	113 ± 17	114 ± 12	.949
*subtest similarities*	114 ± 17	117 ± 13	.465	116 ± 18	117 ± 18	.705
*subtest picture completion*	105 ± 23	113 ± 17	.072	108 ± 20	107 ± 18	.728
*subtest block design*	108 ± 22	110 ± 17	.711	108 ± 18	108 ± 17	.643
*estimated verbal IQ*	115 ± 17	116 ± 13	.937	114 ± 16	115 ± 13	.825
*estimated performance IQ*	106 ± 20	111 ± 15	.143	107 ± 18	108 ± 15	.851
*estimated total IQ*	110 ± 17	113 ± 12	.302	111 ± 16	111 ± 12	.837
GAF, *mean ± SD*^b^	69.4 ± 17.1	74 ± 14.2	.191	75.1 ± 14.7	76.5 ± 15.8	.509
PANSS autism severity score, *mean ± SD*^b^	20.3 ± 7.7	20.1 ± 7.5	.842	13.3 ± 6.5	13.5 ± 6.7	.597
ADOS original algorithm Module 4, *mean ± SD*^b^	11.9 ± 4.1	12 ± 4.3	.962	3.9 ± 4	3.4 ± 3.8	.691
Autism Quotient (AQ), *mean ± SD*^b,d^	22.3 ± 5.9	25.6 ± 4.7	.006	22.8 ± 5.3	25 ± 4.2	.071
Empathy Quotient (EQ), *mean ± SD*^b,e^	18.6 ± 8	15.4 ± 7.6	.044	20.4 ± 10.2	16.7 ± 10.5	.108

*Multiple testing adjusted significances (Bonferroni: p ≤ 0.002) for comparison of each diagnostic group with ASD group are shown: due to missing data upon phenotyping, sample size varies between N = 186-265 in the total sample;* ^a^ *this category includes affective disorders, attention deficit disorder, pervasive developmental disorder not otherwise specified, anxiety disorders, alcohol use disorders;* ^b^ *Mann-Whitney U test;* ^c^ *Chi-square test;* ^d^ *Higher values correspond to more autistic traits;* ^e^ *Higher values correspond to more empathy (i.e. less autistic traits).*

## Results

### PAUSS distribution in the GRAS sample allows for extreme-group definition

The high internal consistency of the autism variables (Cronbach’s alpha: .857; Table 2) indicates their measuring one underlying construct (autism). All item-item correlations were found to be statistically significant (all p-values < .00001, Table 2)*.* Consequently, single PANSS items were summed up to reflect the overall severity of the dimensional trait ‘autism’. The distribution of the *PAUSS* in the schizophrenic GRAS sample encouraged the definition of extreme-groups contrasting maximally with respect to the *PAUSS* (first and last percentile, Figure 1A). Male and female GRAS patients did not differ on the *PAUSS* ratings (Figure 1A). Schizophrenia patients scoring above the last percentile of the *PAUSS* distribution (*PAUSS* 30-52; referred to as ‘autistic schizophrenia patients’) scored higher than ASD patients (mean ± SD: 20.5 ± 7.7) who were more severely impaired than non-autistic schizophrenia (first percentile *PAUSS* 8-10) and disease-control subjects (mean ± SD: 13.3 ± 6.6) on the *PAUSS* (Figure 1B). In terms of sociodemographic and clinical characteristics, the autistic subgroup of schizophrenia patients reached an inferior academic level accompanied by a lower premorbid IQ and had more severe psychopathology and a lower functional status as compared to non-autistic schizophrenia individuals (Table 4). No group differences (autistic versus non-autistic schizophrenia patients) were found with regard to age at onset and duration of disease (Table 4).

### High rate of ASD misdiagnoses in Germany

For *PAUSS* validation, 265 pre-diagnosed adult ASD patiens were recruited throughout Germany. By applying a careful diagnostic procedure (according to DSM-IV-TR criteria) at inclusion in this study, for only 62.3% of patients an ASD diagnoses could be confirmed. The most frequent alternative diagnosis was ‘personality disorders’ (9.8%). A total of 7.2% of the subjects did not fulfil criteria for any psychiatric disorder. For further relevant differential diagnoses compare Table 5. While diverging regarding age at examination, ASD and disease-control patients were comparable with respect to their intellectual functioning (Table 5). Moreover, ASD patients had higher *PAUSS* and ADOS scores than patients with personality and other psychiatric disorders. Interestingly, ASD and psychotic patients did not differ in ADOS and *PAUSS* scores. A higher proportion of ASD patients reported to have no children as compared to disease-controls. Other sociodemographic characteristics like marital or relationship status as well as AQ and EQ were not found to differ between ASD and the overall disease-control sample (Table 5). Compared to the group of participants who did not receive a clinical diagnosis in our study, a larger proportion of ASD patients was single, had no children and was less functional (Table 5). Male and female ASD patients did not differ with respect to sociodemographic characteristics (Table 6).

### Evidence for construct and criterion-based validity of the PAUSS

Construct validity was assessed by evaluating the convergence between *PAUSS* and ADOS. As ADOS scores have been shown to be influenced by age and the intellectual functioning of a given subject [35], we evaluated correlations of ADOS and *PAUSS* with age and the estimated total IQ. Both ADOS and *PAUSS* correlated substantially with age, while only for *PAUSS* a high correlation with the estimated total IQ was found (Figure 2A). Importantly, ADOS and *PAUSS* were found to correlate substantially (r = .763; Figure 2A; partial correlation coefficient when correcting for age and IQ: r = .736, data not shown). All single *PAUSS* items correlated significantly (p < .00001) with the ADOS (Figure 2B). Strongest correlations were observed for ADOS and ‘blunted affect’, ‘social withdrawal’, ‘conversation’ and ‘preoccupation’. ADOS diagnosis groups diverged regarding the *PAUSS* but not regarding AQ and EQ (Figure 2C-E). Individuals who scored above 12 in the ADOS obtained highest, those with a score below 7 lowest *PAUSS* scores. ROC curves for ADOS and *PAUSS* resulted in AuC values of 0.916 and 0.824, respectively (Figure 2F). For AQ and EQ, low AuC values were obtained (0.554 and 0.470, respectively) (Figure 2G). Thus, higher ADOS and *PAUS* scores predicted a higher probability of having a clinical ASD diagnosis whereas AQ and EQ performed at chance level. Cut-off scores and their respective sensitivities and specificities are provided (Table 3). Taken together, evidence for the *PAUSS* to measure autism relevant traits could be obtained.

## Discussion

A multitude of genetic and clinical studies converge on the notion that similar biological pathways may be involved in the etiology of autism and a subgroup of schizophrenia patients [23,39]. Selected items from a clinical rating instrument developed to assess schizophrenia psychopathology (PANSS) were aggregated to characterize autistic symptoms in the GRAS sample of schizophrenic patients *(PAUSS)*. The *PAUSS* was validated in ASD and disease-control samples. *PAUSS* and ADOS correlated substantially. Moreover, the *PAUSS* differentiated between autism (individuals with ASD and autistic schizophrenia subjects), other psychiatric disorders and healthy subjects.

Interestingly, the autistic schizophrenia subgroup had higher *PAUSS* values than the ASD sample. This might be due to the fact that only high-functioning ASD patients, including Asperger’s disorder and autistic disorder patients with average intellectual functioning, were included in the present study (GAF 71, estimated total IQ 111). For the autistic subgroup of schizophrenia subjects functional status and IQ were considerably lower (GAF 31; premorbid IQ 95), and also lower as compared to the non-autistic schizophrenia subjects (GAF 63, premorbid IQ 108). This could point to a generally more affected patient sample where *PAUSS* items reflect the general psychopathology on top of autism-related features.

Over one third of the patients diagnosed or suspected to have autism by healthcare professionals turned out to have a different or no psychiatric diagnosis. Consistent with other reports, common differential diagnoses in the present study were personality and affective disorders [40]. The low reliability of ASD diagnoses in adults may be partially due to the lack of a standardized diagnostic procedure for ASD in adults in Germany [26,41]. Special outpatient centers for ASD in adulthood and specialized practicing psychiatrists are scarce. Owing to the increasing public interest in ASD and the growing demand of diagnostic evaluation [42-44], the procedure has to be efficient. This will enable timely initiation of adequate therapeutic interventions for ASD patients as well as for individuals with other psychiatric disorders. AQ and EQ are often used as screening instruments because they are time-efficient [40]. However, given the broadly impaired introspective capacities of individuals with ASD (e.g. self-referential cognition and empathy) [45,46] and the different degrees of self-evaluation skills acquired by training, in the present study, self-assessment (AQ and EQ) did not differentiate between ASD and differential diagnoses nor did it correlate with ADOS and *PAUSS*. The *PAUSS* in turn may be further psychometrically validated to evolve as a rapid, easy to apply and valid screening instrument ultimately complementing the ADOS.

The findings of the present study are well in line with a previous study illustrating that some patients with treatment-resistant schizophrenia have autistic symptoms and that these co-vary with negative but not with positive symptoms [47]. Notably, this subgroup of patients did not respond to antipsychotic treatment. Neurexin1 *(NRXN1)* is among the genes associated with both ASD and schizophrenia [48,49]. Accumulating evidence reveals that certain *NRXN1* genotypes are overrepresented among non-responders to antipsychotic treatment [50-52]. These reports exemplify the involvement of biological pathways not targeted by conventional dopaminergic agents in a subgroup of schizophrenia patients that likely represents the autistic subgroup.

Prospective research will have to consider whether the phenotypical overlap between autism and schizophrenia shown here indicates that both conditions can emerge from related neurodevelopmental vulnerabilities or shared pathogenic mechanisms based on genotypical overlap [13-15]. It may also address the question whether autism and schizophrenia share other phenotypical aspects, such as neuroanatomical similarities.

## Conclusions

The present work highlights the remarkable convergence of schizophrenia negative symptomatology and autistic features. It shows that the *PAUSS* correlates substantially with the ADOS. Additionally, the *PAUSS* is able to discriminate ASD patients from disease controls. This lends support to the fact that the *PAUSS* is suitable for the dimensional assessment of autistic behaviors in schizophrenia and high-functioning autism. The definition of extreme-groups based on the dimensional *PAUSS* may permit future investigations of genetic constellations modulating autistic phenotypes.
